# Optimal decoding of neural dynamics occurs at mesoscale spatial and temporal resolutions

**DOI:** 10.3389/fncel.2024.1287123

**Published:** 2024-02-14

**Authors:** Toktam Samiei, Zhuowen Zou, Mohsen Imani, Erfan Nozari

**Affiliations:** ^1^Department of Mechanical Engineering, University of California, Riverside, Riverside, CA, United States; ^2^Department of Computer Science, University of California, Irvine, Irvine, CA, United States; ^3^Department of Electrical and Computer Engineering, University of California, Riverside, Riverside, CA, United States; ^4^Department of Bioengineering, University of California, Riverside, Riverside, CA, United States

**Keywords:** neural code, multi-unit activity, averaging, spatial resolution, temporal resolution, hyper-dimensional computing, computational modeling, neural dynamics

## Abstract

**Introduction:**

Understanding the neural code has been one of the central aims of neuroscience research for decades. Spikes are commonly referred to as the units of information transfer, but multi-unit activity (MUA) recordings are routinely analyzed in aggregate forms such as binned spike counts, peri-stimulus time histograms, firing rates, or population codes. Various forms of averaging also occur in the brain, from the spatial averaging of spikes within dendritic trees to their temporal averaging through synaptic dynamics. However, how these forms of averaging are related to each other or to the spatial and temporal units of information representation within the neural code has remained poorly understood.

**Materials and methods:**

In this work we developed NeuroPixelHD, a symbolic hyperdimensional model of MUA, and used it to decode the spatial location and identity of static images shown to *n* = 9 mice in the Allen Institute Visual Coding—NeuroPixels dataset from large-scale MUA recordings. We parametrically varied the spatial and temporal resolutions of the MUA data provided to the model, and compared its resulting decoding accuracy.

**Results:**

For almost all subjects, we found 125ms temporal resolution to maximize decoding accuracy for both the spatial location of Gabor patches (81 classes for patches presented over a 9×9 grid) as well as the identity of natural images (118 classes corresponding to 118 images) across the whole brain. This optimal temporal resolution nevertheless varied greatly between different regions, followed a sensory-associate hierarchy, and was significantly modulated by the central frequency of theta-band oscillations across different regions. Spatially, the optimal resolution was at either of two mesoscale levels for almost all mice: the area level, where the spiking activity of all neurons within each brain area are combined, and the population level, where neuronal spikes within each area are combined across fast spiking (putatively inhibitory) and regular spiking (putatively excitatory) neurons, respectively. We also observed an expected interplay between optimal spatial and temporal resolutions, whereby increasing the amount of averaging across one dimension (space or time) decreases the amount of averaging that is optimal across the other dimension, and vice versa.

**Discussion:**

Our findings corroborate existing empirical practices of spatiotemporal binning and averaging in MUA data analysis, and provide a rigorous computational framework for optimizing the level of such aggregations. Our findings can also synthesize these empirical practices with existing knowledge of the various sources of biological averaging in the brain into a new theory of neural information processing in which the *unit of information* varies dynamically based on neuronal signal and noise correlations across space and time.

## Introduction

Neural dynamics span across a wide range of spatiotemporal scales, from (sub)cellular to regional and from (sub)millisecond to circadian and higher (Buzsaki, 2006; Bressler and Menon, 2010; Breakspear, 2017). Arguably, the most common link between neural dynamics across different spatiotemporal scales is averaging. Macroscopic measurements such as EEG, MEG, and fMRI reflect spatially-averaged activities of millions of neuronal post-synaptic potentials (Logothetis et al., 2001; Buzsáki et al., 2012) which are themselves the result of pre-synaptic spatial averaging through dendritic trees (Cash and Yuste, 1999) and are linked to higher-frequency spiking activity through synaptic temporal averaging (Kandel et al., 2013). Averaging is also the theoretical foundation for the broad family of mean-field models (Buice and Cowan, 2009; Breakspear, 2017), and is further applied across imaging modalities as a signal-processing step *for improving signal to noise ratio (SNR)* (Poldrack et al., 2011; Luck, 2014; Widmann et al., 2015). Averaging or averaging-involved methods such as spatial smoothing and parcellation of voxel-wise fMRI, low-pass filtering, principal component analysis (PCA), independent component analyses (ICA), peri-stimulus time histograms (PSTH), and firing rate estimations are all popular means for reducing the dimensionality of data and making large-scale brain recordings understandable and explainable.

On the other hand, averaging also involves an inevitable loss of information. This can be seen, at a generic level, from the information-theoretic data processing inequality (Cover, 1999). In a series of recent works (Ahmed and Nozari, 2022, 2023; Nozari et al., 2023), we have further shown that averaging has a particularly strong linearizing effect, transforming functionally-relevant nonlinearities (spiking, multi-stability, limit cycles, etc.) into what appears to be “noise” in macroscopic measurements. Notably, *the strength of this linearizing effect is directly related to the amount of signal correlation among the averaged units*: the higher signal correlation is among a group of neurons and the slower it decays with distance between them, the weaker the linearizing effect of averaging becomes, i.e., the more neurons we need to average over before nonlinearities fade (Nozari et al., 2023).

As such, averaging can have a dual effect on the neural code: it can improve SNR by averaging over noise, but it can also degrade SNR by canceling out functionally-relevant nonlinearities. The balance of these two effects depends on the relative strength of signal and noise correlations among neurons. *If* noise correlations are weaker and decay more rapidly with distance, then controlled amounts of averaging can be beneficial by canceling noise faster than fading the signal. Otherwise, no amount of averaging would be beneficial and the neural code can be best decoded from the raw spiking activity of individual neurons with millisecond resolution.

In this work, we test the *central hypothesis* that there exists an optimal amount of spatial and temporal averaging, i.e., an optimal spatiotemporal resolution, which maximizes neuronal SNR and therefore the accuracy of decoding the neural code. Using data from *n* = 9 mice from the Allen Institute Visual Coding - Neuropixels dataset, we design computational models that classify visual images shown to each mouse using its large-scale MUA with parametrically varied amounts of spatial and temporal averaging. The use of the brain-inspired hyper-dimensional computing (HDC) framework (Kanerva, 2009; Schlegel et al., 2022; Zou et al., 2022) allows us to gain precise control over the amount of end-to-end spatiotemporal averaging performed by the decoder and minimize implicit sources of averaging that extensively occur during the training of most machine learning alternatives and can confound our findings. The resulting HDC-based classifier, termed NeuroPixelHD, provides a means to testing this work's central hypothesis as well as a general-purpose model for encoding and decoding large-scale MUA data in a transparent and interpretable manner owing to the symbolic nature of HDC.

## Results

### NeuroPixelHD: a hyperdimensional model for large-scale multi-unit activity

In this work we use the brain-inspired framework of HDC (Kanerva, 2009; Schlegel et al., 2022; Zou et al., 2022) to design NeuroPixelHD, an efficient decoding model for MUA. The use of HDC to test our central hypothesis is motivated by the core observation that vector summation results in an *irreversible averaging* in small dimensions (i.e., the summands are not recoverable from the sum), but it can result in *reversible memorization* in very large dimensions (Supplementary Note 1). As such, a trained HDC model can embed a copy of all of its training samples, without any unintended implicit averaging. In this work, we train NeuroPixelHD to classify images within two categories based on MUA recordings: Gabor patches at different locations of a 9x9 grid in the visual field, and 118 different images of natural scenes (Methods).

**Figure 1:**
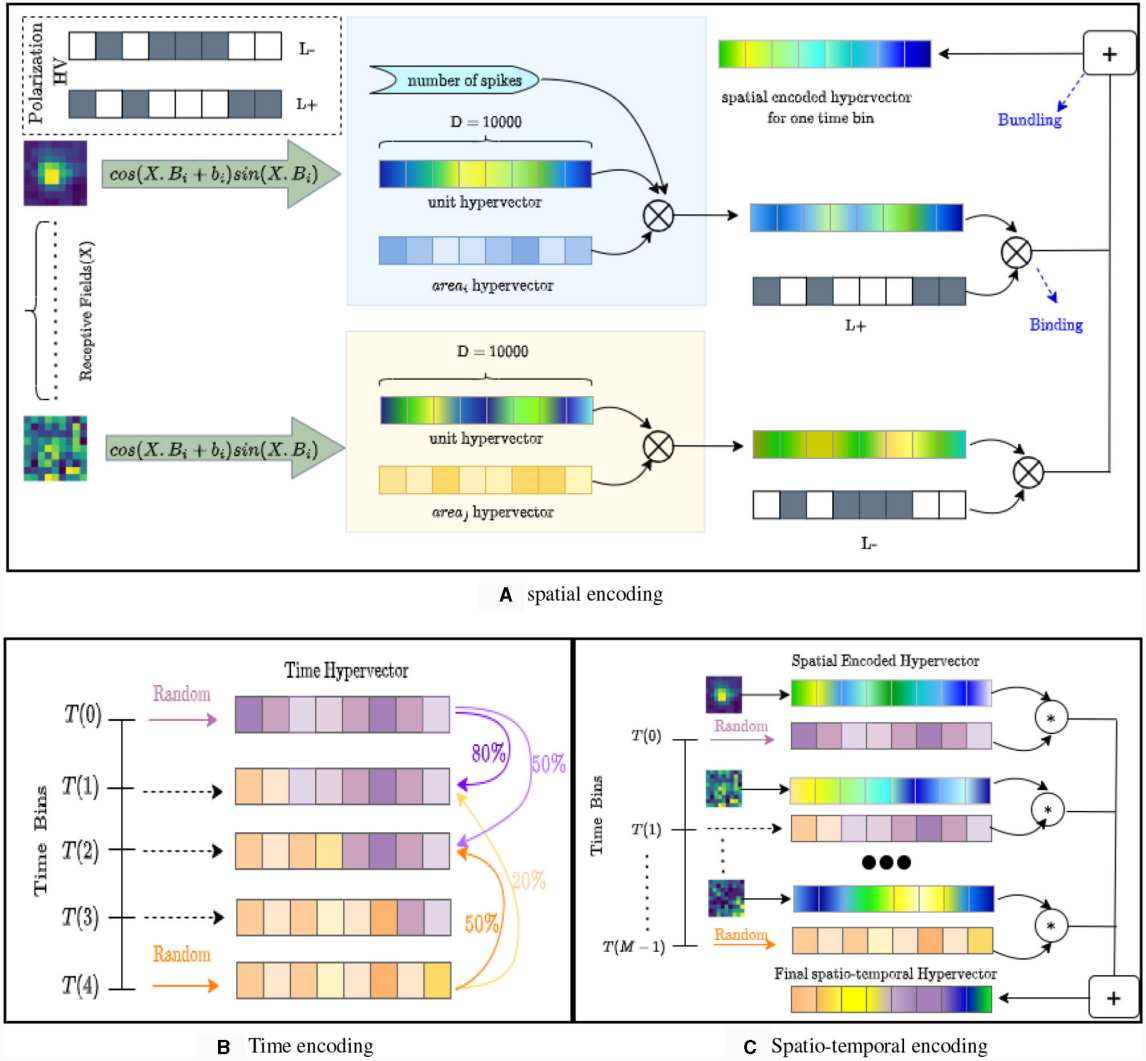
The structure and encoding of NeuroPixelHD. **(A)** Spatial encoding in NeuroPixelHD. Spatially correlated hypervectors are generated for each neuron, using each neuron's responses to receptive field tuning and cosine encoding, and then bound with (randomly generated) hypervectors representing corresponding brain areas. **(B)** Temporal encoding in NeuroPixelHD. Two random hypervectors are generated for times 0 and 250 ms and then linearly interpolated, via dimension borrowing, to generate correlated hypervectors for intermediate time points. **(C)** Encoding of each trial in NeuroPixelHD.

Inspired by our earlier work on event-based cameras (Zou et al., 2022), the design of NeuroPixelHD involves an encoding phase and an adaptive training phase. During the encoding phase, the binned spike counts of all the recorded neurons throughout each trial (250 ms here) is encoded into one hyper-vector (HV) (Figure 1). As described in details in Methods, The encoding involves a sequence of reversible binding and bundling operations (standard in HDC, see Methods) over three ingredients: binned spike counts, neuron HVs, and time bin HVs. Neuron HVs are generated based on each neuron's anatomical region and response during receptive field tuning, therefore maintaining a level of spatial correlation proportional to the anatomical and functional proximity of each pair of neurons. Time bin HVs are generated randomly for the beginning and end of each trial (0ms and 250 ms) and interpolated via linear dimension borrowing for intermediate bins, maintaining a level of temporal correlation proportional to the temporal proximity of each pair of time bins. These HVs are then fused with binned spike counts using binding and bundling operations to encode all the neural activity during each trial into one trial activity HV used during the second phase for classifier training.

The second phase of NeuroPixelHD consists of adaptive training. Each class (Gabor location or natural scene image) is represented by one class HV. All class HVs are initialized at zero and iteratively updated such that the similarities between each class HV and corresponding trial activity HVs gradually increase and the similarities between each class HV and trial activity HVs of other classes gradually decrease (see Methods). We use cosine similarity (normalized dot product) in NeuroPixelHD due to its simplicity and computational efficiency, but various other measures of similarity have also been proposed in HDC and can be alternatively used. At the end of training, each test trial is assigned to the class that has the largest similarity between its class HV and activity HV of that test trial. To measure classification accuracy, we use standard F1 score for natural scene images and median Euclidean error between the actual and predicted locations for Gabor patches (see Section Methods).

### 125ms temporal resolution maximizes visual decoding accuracy for static images

We investigated the optimal amount of temporal averaging for visual decoding by comparing the decoding accuracy of NeuroPixelHD for varying bin size values. We started from the smallest bin size of 1 ms and gradually increased the bin size until reaching one bin for the entire trial duration (250 ms). As we increase the bin size, both the signal and the noise components of spike counts are averaged, potentially changing the spike counts' signal to noise ratio and, in turn, the decoding accuracy of the downstream classification.

**Figure 2:**
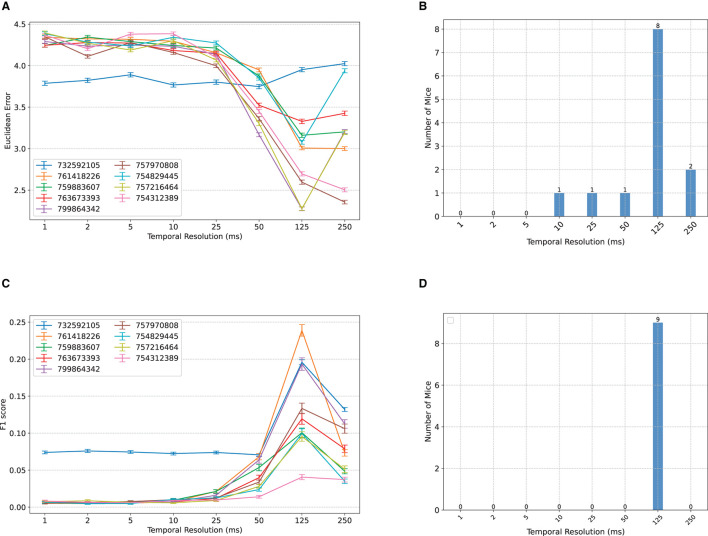
Comparisons between classification accuracy of NeuroPixelHD with different temporal resolutions. **(A)** Mean Euclidean distance errors of NeuroPixelHD in classifying Gabor locations. Each line corresponds to one mouse (*n* = 9) and error bars represent 1 s.e.m. **(B)** Distribution showing the number of mice for whom each time bin is optimal. The optimal time bin for each mouse was selected based on Wilcoxon signed rank test with α = 0.05. In cases where 2 or more bin sizes had the least error (insignificant statistical difference), all of them were counted as optimal bin size for that mouse and included in the aggregate bar graph. **(C, D)** Similar to **(A, B)** but for the classification of nature scenes. Here accuracy is measured by F1 score (higher is better; see Methods). Across both tasks, the 125 ms time bin resulted in maximum decoding accuracy.

When classifying the location of Gabor patches from binned MUA spike counts, in most subjects, we observe an initial insensitivity of classification accuracy to bin size between 1–10 ms, followed by a sharp improvement in decoding accuracy until 125 ms, and an occasional worsening of accuracy afterwards (Figure 2). Remarkably, for most subjects, the worst accuracy occurs at the smallest bin size, despite the classifiers' access to *all* spike count information. This may be at first counter-intuitive from an information-theoretic perspective [cf., e.g., the Data Processing Inequality (Cover, 1999)], but demonstrates the importance of optimal feature extraction from a machine learning perspective and is consistent with the common perception of individual spikes as being highly noisy and the common practice of binning spike counts before using them for downstream analyses.

To further resolve the heterogeneity among subjects and compute the optimal temporal resolution at the group level, we found the optimal bin size for each subject (namely, the bin size with the lowest median classification error) and calculated, for each bin size, the number of subjects for whom that bin size is optimal. If two or more bin sizes were jointly optimal (*p* ≥ 0.05, Wilcoxon signed rank test), we included all of them in the group-level count. The result, shown in Figure 2B, corroborates that 125ms resolution is optimal at the group level, 250 ms is the second best, and 1-5ms resolution yields the least signal to noise ratio overall. The same trend appears even more contrastively for the decoding of natural scenes (Figures 2C, D). Here, we measure classification accuracy using F1 score with higher values indicating higher accuracy. Across all subjects, the 125ms resolution provides the highest decoding accuracy, while the 1–10 ms resolutions result in chance level classification (1/118 ≃ 0.008) in all but one mouse.

We also investigated a more precise quantification of optimal temporal resolutions by including all regularly spaced bin sizes with a 25ms increment (i.e., 75, 100, 150, 175, 200, and 225 in addition to the original selection). The newly added bin sizes are harder to interpret and are generally avoided in this study since each spike can no longer be included in exactly 1 bin, thereby making their comparison against bin sizes that are divisors of 250 ms potentially unfair. However, we mitigated this potential unfairness as much as possible by computing the decoding accuracy of NeuroPixelHD at the newly added time bins via two methods and averaging the results: overlapping, whereby time bins are allowed to overlap in order to cover the whole 250ms duration of each trial (over-counting), and cropping, whereby time bins are not allowed to overlap and some remaining portion of the 250 ms duration is discarded accordingly (under-counting). The results are shown in Supplementary Figure S4. Interestingly, while 125ms still remains optimal for most subjects across both tasks, a distinction now appears between the two tasks: Gabor locations are encoded at slightly slower resolutions than the identity of natural scene, even though both categories of images are displayed for the same duration (250ms) and alternated without any inter-stimulus delay. This result provide evidence that even within the same sensory modality and task structure, stimulus content and complexity can affect the resolution at which neural information is encoded.

### Optimal temporal resolution follows a top-down hierarchy and is significantly correlated with theta oscillations

**Figure 3:**
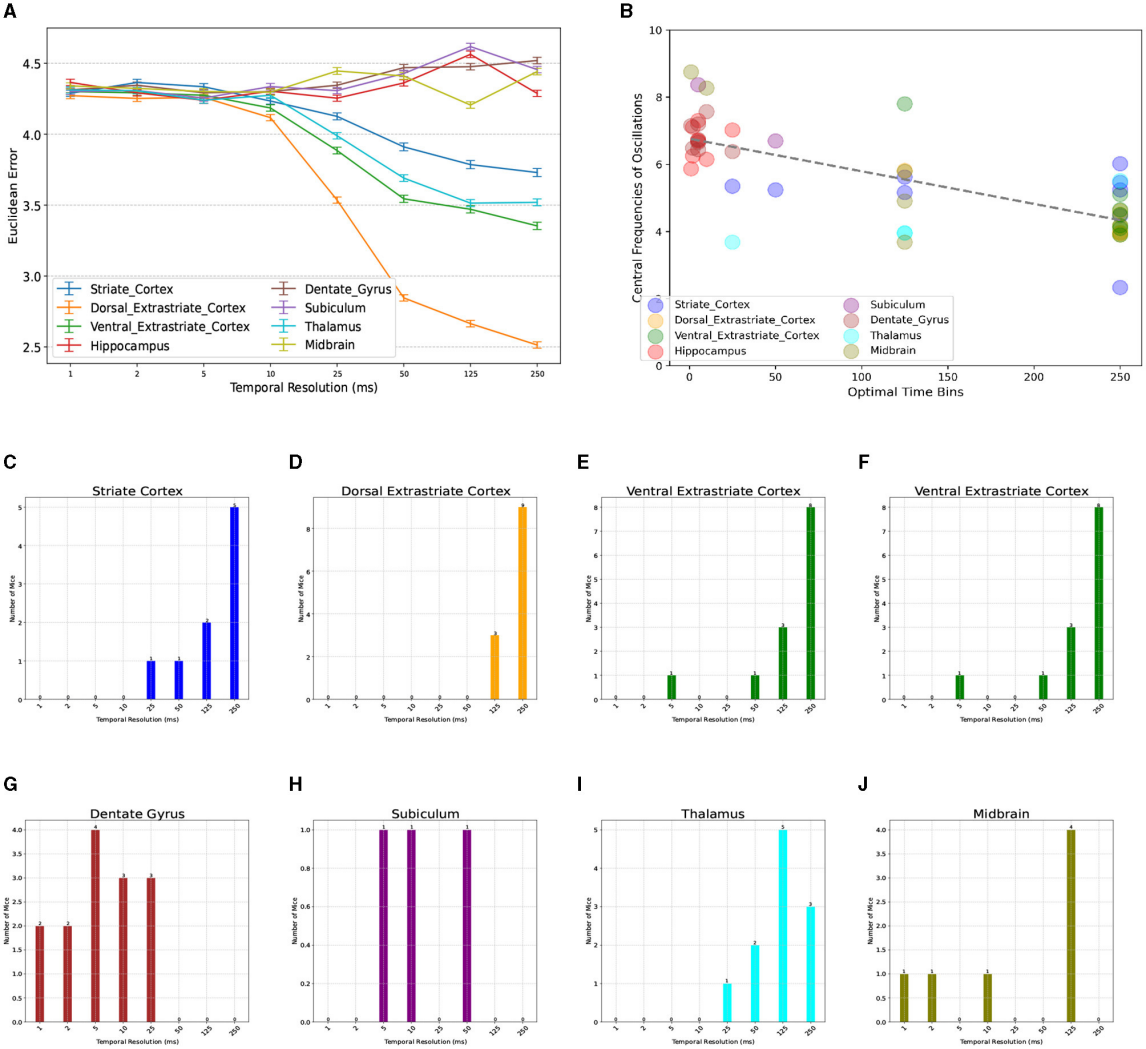
Breakdown of optimal temporal resolution by brain region and its relationship with neural oscillations. **(A)** Similar to Figure 2A but averaged over mice and distinguished across brain regions. On average, thalamic and visual areas have the largest optimal resolutions (250 ms), whereas regions across the hippocampal formation have significantly smaller optimal resolutions (1–25 ms). Midbrain areas show more variable patterns but most often prefer 125 ms. A breakdown of optimal resolutions across regions and mice can be seen in **(C–J)**. Power Spectrum Across Various Brain Regions. **(B)** Relationship between regionally-optimal temporal resolutions and the central frequency of each region's neural oscillations across all mice (*r* = −0.73, *p* < 10^−4^, randomization test) (see Methods). Regions with slower oscillations, particularly within the theta range, have longer optimal temporal resolutions and vice versa.

We next investigated the consistency of this optimal temporal resolution across the available brain regions (Table 1). Neuronal dynamics of different brain regions are known to have a hierarchy of *time constants*, whereby the autocorrelations of signals recorded from lower-level sensoritmotor areas decays faster with lag than those recorded from higher-level association cortices (Murray et al., 2014). To test whether a similar pattern exists in the optimal temporal resolution of distinct regions, we compared the accuracy of NeuroPixelHD when using binned spike trains of areas within only one brain region at a time (Figure 3A). We found a wide variation in the optimal temporal resolutions across regions, ranging (on average) from 5-250ms. Furthermore, we observed a spatially-organized and hierarchical pattern in the regionally-optimal resolutions, whereby visual areas (both early and later) have the slowest resolution (250ms) and areas across the hippocampal formation have the fastest resolutions (~5ms on average). Thalamus and midbrain areas most often prefer the globally-optimal 125ms resolution, though the latter shows little sensitivity to temporal resolution in general (Figures 3C–J). Therefore, the globally-optimal 125 ms resolution has arisen from and should be understood as a trade off between a top-down hierarchy of regions that encode information at significantly slower and faster resolutions.

**Table 1:** List of brain areas available within each brain region.

**Region**	**Included areas**
Striate cortex	VISp
Dorsal extrastriate cortex	VISam, VISal, VISrl, VISmma
Ventral extrastriate cortex	VISpm, VISl
Hippocampus	CA1, CA2, CA3
Subiculum	SUB, ProS
Dentate Gyrus	DG
Thalamus	TH, LP, LGv, LGd, PP, PIL, MGv, PO, Eth, POL
Hypothalamus	ZI
Midbrain	APN

Each mouse has data from neurons in some subset of these areas. The “dorsal” and “ventral” extrastriate cortices are named as such for ease of reference and based on homology to the primate brain (Marshel et al., 2011), not anatomical location in the mouse brain [cf. Allen Institute (2019), Figure 6].

Given the long history of neural oscillations across the studied regions and the hypothesized role of oscillations in information transfer, we next tested whether the observed regional and subject-to-subject variability in optimal temporal resolutions is related to ongoing neural oscillations. For each mouse and brain region, we first computed the average firing rate of all neurons within each region (binned at 1ms) and used the FOOOF toolbox (Donoghue et al., 2020) to find the central frequency of the slowest oscillation in each case (see Methods for details). Indeed, we found a strong relationship between the resulting central frequencies of ongoing oscillations and optimal temporal resolutions, whereby regions with slower ongoing oscillations, particularly in the theta (4-8Hz) range, tend to also encode information at proportionately slower temporal resolutions (Figure 3B, Pearson *r* = −0.73, *p* < 10^−4^, randomization test). This finding suggests theta-band neural oscillations as a partial mechanistic explanation of the functionally-discovered optimal temporal resolutions via NeuroPixelHD, while additional investigations are needed to fully uncover the biological mechanisms underlying neuronal information encoding at distinct resolutions.

### Population and area level spatial resolutions maximize visual decoding accuracy

We next performed a dual analysis, comparing the visual decoding accuracy of NeuroPixelHD when the spike counts provided at its input were spatially averaged at progressively larger scales. We used five levels to divide the range from micro to macro scale: single neuron level, where no averaging is performed; population level, where the spike counts of regular spiking (putatively excitatory) and fast spiking (putatively inhibitory) neurons within each brain area were combined; area level, where the spike counts of all neurons within each area were combined; region level, where the spike counts of all neurons within all areas of each brain region were combined; and whole-brain level, where the spike counts of all recorded neurons were combined (cf. Methods). The classification accuracy of NeuroPixelHD was then compared between these levels for each mouse, separately for the Gabor patches and natural scenes.

**Figure 4:**
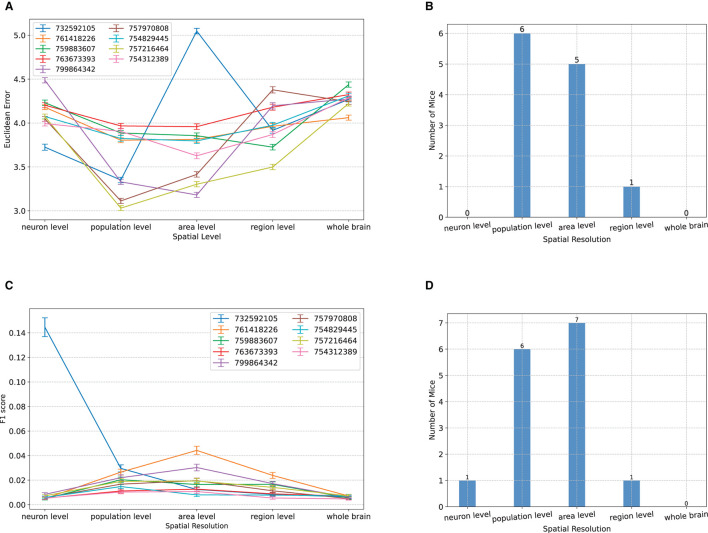
Comparisons between classification accuracy of NeuroPixelHD with different spatial resolutions. **(A–D)** Panels parallel those in Figure 2 except that averaging is performed over space (clusters of neurons). See Methods for a description of each spatial scale. All comparisons are performed without temporal averaging (1ms time bin). Across both tasks and most subject, either the population level or the area level resolutions led to maximum visual decoding accuracy.

Similar to the above analysis of temporal averaging, the optimal resolution was at the micro nor at the macro scales, but rather at an intermediate (meso) scale. For the classification of the spatial location of Gabor patches, for almost all subjects, maximum decoding accuracy (minimum Euclidean error) was obtained at either the population level or the area level (Figure 4A). In particular, the two extremes of neuron and whole-brain levels are significantly worse than the intermediate levels and not optimal in any of the subjects (Figure 4B). The same trend also appeared in the decoding of images of natural scenes. For all but one subject, NeuroPixel's classification accuracy (measured via F1 score, see Methods) was at the chance level $\frac{1}{118}=0.008$ at the neuron and whole-brain levels and reached its maximum at an intermediate level (Figure 4C). In fact, in most subject, the maximum decoding accuracy was obtained at either the population or the area level as was the case in the Gabor task (Figure 4D).

In summary, across both the temporal and spatial dimensions of visual coding as well as spatial localization and object identification, we found intermediate resolutions, rather than the micro or macro extremes thereof, to maximize decoding accuracy in most cases. This is consistent with a model in which noise correlations decay more rapidly among nearby neurons than do signal correlations, and confirms our initial hypothesis that averaging initially improves, but then degrades, neuronal SNR and therefore the accuracy of decoding the neural code.

### NeuroPixelHD has similar accuracy to other machine learning classifiers but unique structure for unbiased detection of optimal resolutions

**Figure 5:**
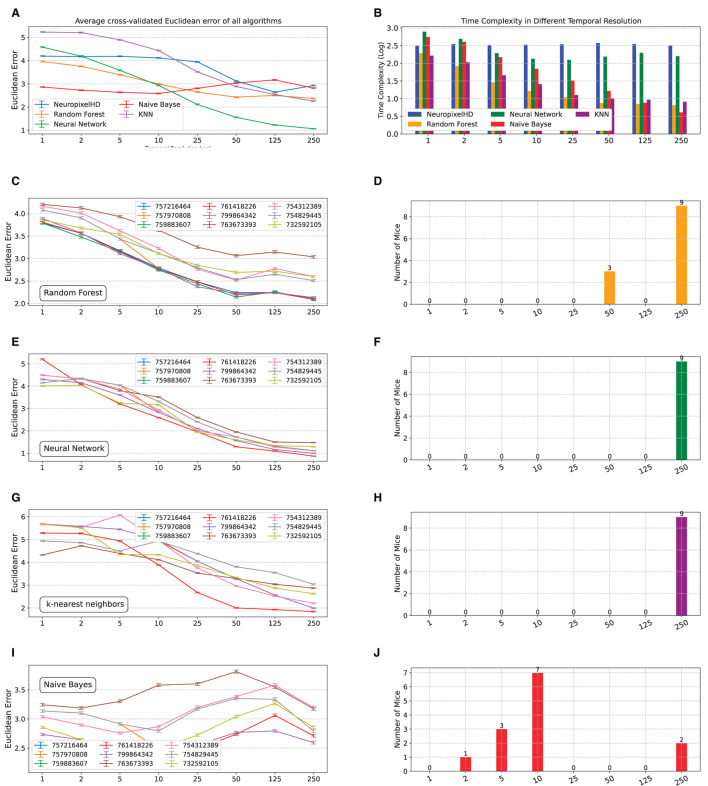
Comparing NeuroPixelHD with alternative machine learning classifiers. We compared the accuracy and time complexity of NeuroPixelHD with random forest (RF), artificial neural network (ANN), K-nearest neighbor (KNN), and naive Bayes (NB) classifiers at different temporal resolutions. All models are trained for classifying the location of Gabor stimuli (cf. Methods) at the neuron level spatial resolution, separately for *n* = 9 mice **(A)** Average (across mice) cross-validated accuracy (Euclidean error) of all algorithms at different temporal resolutions. NeuroPixelHD has comparable accuracy with other algorithms. **(B)** Similar to **(A)** but for the time complexity of different algorithms. The time complexity of NeuroPixelHD is comparable to other algorithms at the 1 ms resolution where they have comparable input dimensions (cf. Methods), but remains flat with resolution unlike other algorithms that generally become more efficient at coarser resolutions. **(C–J)** Similar to Figures 2A, B for RF, KNN, ANN, and NB, respectively.

We next compared NeuroPixelHD against alternative state-of-the-art machine learning classifiers, namely, random forest, artificial neural networks, K-nearest neighbor (KNN), and naive Bayes (see Section Methods). In general, we found the decoding accuracy of NeuroPixelHD to be comparable with other algorithms and significantly better than chance at its optimal resolutions (Figure 5A, Supplementary Figure 5A), ensuring its viability as a normative decoding algorithm as used in this study. Similarly, NeuroPixelHD has comparable time complexity relative to other algorithms at fine resolutions, but its time complexity remains flat with resolution whereas other algorithms often become faster at coarser resolutions (Figure 5B, Supplementary Figure 5B). These findings are in line with prior findings in the HDC literature (Hernández-Cano et al., 2021) and highlight the need for targeted application of HDC-based models. In other words, HDC-based models such as NeuroPixelHD are not universally better or worse than other algorithms, but are particularly beneficial for applications that benefit from the symbolic architecture of HDC and the implications thereof (such as averaging-free operations, brain-inspired encoding, transparency, etc).

Despite having similar overall accuracy, NeuroPixelHD and other algorithms give rise to distinct optimal temporal resolutions (Figures 5C–F, Supplementary Figures 5C–F). This can be at least due to two main sources of bias in the alternative algorithms with regards to detecting optimal resolutions: implicit averaging and input dimensionality. As noted earlier, averaging occurs in many forms during the encoding and training of many machine learning classifiers and can make them need less explicit averaging to optimize their accuracy, therefore biasing their optimal resolutions toward finer scales. In the naive Bayes classifier, e.g., averaging is the key operation in computing the mean and variance of Gaussian likelihoods from training samples, and is likely contributing to the fact that naive Bayes classification reaches its maximum accuracy in decoding Gabor locations at 10ms for most mice (Figures 5I, J).

In most other cases, however, an opposite bias toward coarser resolutions seems to be dominant in the alternative classifiers (Figures 5C–H, Supplementary Figures 5C–J). Coarser resolutions lead to lower-dimensional inputs (features), which are preferred by many machine learning models particularly when learning patterns from small amounts of training data, as is often the case in neural recordings. This can bias the resulting optimal bin size toward coarser resolutions even if more information is present in higher-dimensional inputs corresponding to finer resolutions. This source of bias seems to be frequently dominating other machine learning alternatives, all of which often prefer the coarsest temporal resolution (250ms). In NeuroPixelHD, however, data at all resolutions are mapped into the same hyperdimension, preventing such dimensionality-induced bias toward finer or coarser resolutions.

Finally, we examined the *interplay* between temporal and spatial resolutions by comparing the decoding accuracy of NeuroPixelHD and alternative machine learning methods across different temporal (spatial) resolutions while the spatial (temporal) resolution is optimized. In both cases, we expect less averaging to be required in one dimension (temporal or spatial) for reaching optimal accuracy, compared to the case when the other dimension was at its finest level (Figures 2, 4), as both dimensions of averaging ultimately improve signal to noise ratio through the same underlying mechanism (diminishing noise faster than diminishing signal). This was indeed the case both spatially (Supplementary Figures S6, S7) and temporally (Supplementary Figures S8, S9) and in nearly all algorithms, although to different degrees. All algorithms, except for naive Bayes, prefer neuron-level spatial resolution when decoding at mouse-specific optimal temporal resolution, and prefer a finer temporal resolution when decoding at population-level spatial resolution, exhibiting a consistent pattern of inter-dependence between averaging dimensions whereby finer resolutions in one dimension necessitate coarser resolutions in the other and vice versa.

## Discussion

In this study we designed NeuroPixelHD, a normative hyperdimensional computational model for large-scale MUA, and used it to probed into the effects of spatial and temporal averaging on the neuronal signal to noise ratio in the brain. While the largely averaging-free architecture of NeuroPixelHD was its key property in allowing us to gain precise control over the amount of end-to-end averaging performed by the model and achieve an unbiased detection of optimal resolutions, we also demonstrated its comparable accuracy and efficiency compared to several alternative machine learning models. We compared the decoding accuracy of NeuroPixelHD from large-scale MUA when its input spike counts were averaged to varying degrees over space and time. We found 125ms temporal resolution and population-area level spatial resolution to maximize, on average across the whole brain, the accuracy of decoding both the spatial location of Gabor patches and the identity of natural scenes. We further observed a broad hierarchy of finer time resolutions, significantly modulated by the central frequency of theta-band oscillations, across different brain regions. Finally, we observed an interplay between optimal spatial and temporal resolutions, whereby increasing the amount of averaging across one dimension (space or time) decreases the amount of averaging that is optimal across the other dimension, and vice versa.

The globally-optimal resolution of 125ms, as well as the wide range of locally-optimal resolutions observed in Figure 3, reflect the interplay of various mesoscale dynamics across the brain. It is well-known that these mesoscale dynamics are distinct from, even though highly intertwined with, the microscale processes of spike generation. The latter involve sub-millisecond dynamics up to about 5KHz or even higher, whereas the primary focus of our study is the significantly slower dynamics of spike interpretation and decoding. Also, even at the mesoscale, population dynamics involved in visual processing are well-known to rely on dynamics faster than 125ms, such as gamma oscillations (Eckhorn et al., 1988; Gray et al., 1989; Fries et al., 2001). While these fast dynamics are likely critical for generation and successful transfer of spikes between cortical columns involved in processing the same visual features and dimensions (Fries, 2005) [i.e., at the implementational level (Marr and Poggio, 1976)], they do not necessarily imply that a downstream area seeking to decode the external visual scene from such spike streams must do so at equally fast resolutions [i.e., at the algorithmic level (Marr and Poggio, 1976)]. Our findings shed light on the latter, suggesting that streams of spikes evoked by static visual stimuli in various brain regions are sufficiently stationary over intervals of approximately 250ms in visual cortex, 125ms in the thalamus, and 5ms in hippocampal areas such that averaging spike streams over such intervals effectively preserves the signal while minimizing noise.

Mechanistically, implementation of such an optimal decoding at a downstream region, such as higher-level association cortices, requires a neurobiological mechanism for low-pass filtering of spikes at this resolution. Synaptic transmission provides a natural mechanism for this purpose. NMDA Glutamate receptors, GABA-A receptors, and Nicotinic ACh receptors all have time constants close to 125 ms (Jones and Bekolay, 2014). These receptors are well-known to mediate various functions, including synaptic plasticity, regulation of excitability, and attention which are all relatively slow and can all benefit from such integration of spikes and improvement in spiking signal to noise ratio.

An interesting finding of this study was a confirmation of the widespread belief that neural populations clustered based on cell type form functionally relevant units for studying the neural code (Klausberger and Somogyi, 2008; Pfeffer et al., 2013; Jadi and Sejnowski, 2014). However, our results also show that in the absence of ground-truth genetic information, this is a nuanced clustering sensitive to the functional proxy used for cell type differentiation. Putatively excitatory and inhibitory neurons are often interchangeably classified based on their spiking waveform shape or spiking statistics (Connors and Gutnick, 1990; Barthó et al., 2004; Becchetti et al., 2012; Tseng and Han, 2021). However, we found the two proxies to lead to notably distinct clusters (Supplementary Figure 1B) and clustering based on Fano factor to give significantly better classification results (Supplementary Figures 1C, D). This marked difference is in need of further mechanistic investigation, but in itself highlights the importance of functional proxies used for population-level analysis of neural dynamics.

An unconventional aspect of NeuroPixelHD encoding is the use of independent time bin HVs for different trials (even though time bin HVs within each trial are correlated, cf. Methods). This is critical for preventing averaging to occur among trial HVs during the adaptive training process where HVs of different trials are linearly combined (bundled). Using shared time bin HVs would instead result in every pair of trial HVs to become more similar to each other, due to the shared spatial and magnitude similarity within the same time bin. This is the similarity preserving property of binding: δ(*a* ⊗ *c, b* ⊗ *c*) = δ(*a, b*). In comparison, When independent time bin HVs are applied, the same similarity no longer transfers to similarity between trial HVs, leading to a broader and more widespread usage of the hyperspace (cf. Supplementary Note 1). On the other hand, using *shared* time HVs leads to an improved classification accuracy (Supplementary Figure S2). This is expected, particularly in light of the benefits of moderate amounts of averaging that we observed (cf. Results), but is still undesirable for the purposes of this study as the implicit averaging implied by using shared time bin HVs can confound the explicit amounts of averaging we perform at each spatiotemporal resolution and potentially bias our results.

A note is also warranted on our use of HDC (as opposed to other machine learning architectures) in designing NeuroPixelHD. From a purely machine learning perspective, HDC-based models are often sought for their transparency and interpretability (Imani et al., 2021; Thomas et al., 2021; Kleyko et al., 2023) while they may also at times achieve higher task accuracy (Imani et al., 2017; Kim et al., 2018) and/or computational efficiency (Ge and Parhi, 2020) compared to non-symbolic alternatives. However, the key advantage of HDC in the present study is its averaging-free nature. Testing our central hypothesis, i.e., that there exists some intermediate amount of averaging which is optimal for decoding the neural spiking information, necessitates using a computational framework that refrains from implicit averaging of sample inputs during training. HDC not only affords this property, but does so in a brain-inspired way (as opposed to, e.g., k-nearest neighbor classification that is also averaging free but completely non-biological). In this context, the transparency and interpretability of HDC further act as “bonus" characteristics that can potentially be leveraged in future studies for gaining a deeper understanding of neural processing. Finally, we should emphasize that NeuroPixelHD is the first HDC-based classifier designed for MUA data, and thus may not be the best one. Future studies are needed to investigate the full potential of HDC in encoding and decoding large-scale MUA.

This study has a number of limitations. As noted earlier, NeuroPixelHD is not necessarily the best HDC architecture for encoding and decoding large-scale MUA data. Our analysis is further limited to two categories of static visual stimuli, making it possible that other, possibly very different, spatial and temporal resolutions are optimal for different categories of stimuli, sensory modalities, and tasks. Notably, the globally-optimal 125 ms temporal resolution we found is equivalent to an 8Hz sampling rate or a 4Hz Nyquist frequency, which is also the frequency at which the visual stimuli are shown in this experiment (each stimulus lasting 250 ms). This can suggest a testable hypothesis for further investigation, namely, that the optimal temporal resolution for detecting any stimulus depends on the dominant frequencies present in that stimulus. Faster sampling may not provide a significant advantage, while averaging at frequencies close to stimulus band-width can improve signal quality by averaging out other (irrelevant) variations. For other tasks, such as viewing natural movies or drifting gratings with varying frequencies, and other sensory modalities, this hypothesis would predict the optimal temporal resolution to become faster as the bandwidth of the *stimulus dynamics* increases (involves higher frequencies). Moreover, our analyses of optimal spatial resolution is likely confounded by the sparse sampling of neurons in our dataset. Should we had access to spiking activity of all neurons in each region, we might have found different, possibly finer, resolutions to be optimal for decoding. Finally, further studies are needed to confirm the generalizability of our findings to humans and other species.

Overall, this study presents empirical support for the presence of an optimal amount of spatial and temporal averaging that maximizes the neuronal signal to noise ratio, and provides an initial estimate of optimal spatial and temporal resolutions during passive viewing of static images. Future work is needed to extend these estimates to other tasks, sensory modalities, and species. While about half of the variance of optimal temporal resolutions across mice and brain regions was explained by theta-band central frequency, further investigations are necessary to more accurately explain our data-driven optimal resolution estimates, potentially by linking them to underlying biological mechanisms such as synaptic time constants noted earlier, axonal conduction velocities, and signal and noise correlations among populations of excitatory and inhibitory neurons. Axonal conduction velocities putatively affect optimal temporal resolutions as they regulate the time that it takes for spikes from one region to travel to another. Thus, when projections from multiple regions converge on a downstream region, coordination would be essential for the post-synaptic potentials (PSPs) resulting from one stream of spikes to be able to efficiently interact with the PSPs from other streams. Longer optimal time windows, such as those in the visual cortex and thalamus, would then provide for a relatively broad window of time during which the accumulation and averaging of spikes can happen and result in an efficient decoding. In contrast, shorter windows such as those observed in hippocampal areas make the precision of conduction velocities (regulated by glia) much more critical. Further, our results have clear implications about relative signal and noise correlations among populations of excitatory and inhibitory neurons. In the majority of our subjects, the population level spatial resolution was either optimal or nearly so. In light of our earlier results [see, e.g., Figure 4 of Nozari et al. (2023)], this optimally of population-level spatial resolution suggests that within the same population of neurons, there exists a significantly stronger signal correlation than noise correlation, making averaging over each population beneficial for decoding accuracy. Averaging over larger spatial scales gradually loses this benefit, perhaps due to a weaker signal correlation at larger scales.

Finally, it remains an invaluable area of future research to understand the relationship between the spatiotemporal resolutions that are optimal for a normative decoding model such as NeuroPixelHD and those that are optimal for and/or employed by the brain itself.

## Materials and methods

### Visual coding—neuropixels dataset

In this study, we utilized data from the Allen Brain Observatory, specifically from experiments conducted with Neuropixel probes in wild-type mice. The initial Neuropixels data release encompassed responses from neurons in the visual cortex, hippocampus, and thalamus, including brain regions such as: Striate Cortex, Dorsal Extrastriate Cortex, Ventral Extrastriate Cortex, Hippocampus, Subiculum, Dentate Gyrus, Thalamus, Hypothalamus, and Midbrain.

Different visual stimulation tasks were administered to mice, as illustrated in Figure 4. However, for our data analysis, we focused on two specific tasks: Gabor and natural scenes. All experimental sessions commenced with a receptive field mapping stimulus. During the Gabor task, Gabor patches were randomly displayed at one of 81 locations on the screen, forming a 9 × 9 grid. Each patch appeared for 250 ms, without any blank intervals, and this process was repeated 45 times for each location.

For the natural scenes task, a stimulus comprising 118 grayscale natural images was employed. These images were sourced from the Berkeley Segmentation Dataset (Martin et al., 2001), the van Hateren Natural Image Dataset (Van Hateren and van der Schaaf, 1998), and the McGill Calibrated Color Image Database (Olmos and Kingdom, 2004). Prior to presentation, the images underwent contrast normalization and resizing to 1,174 × 918 pixels. Each image was randomly shown for 0.25 seconds, without any intervening gray period. For this task, each image was shown 50 times.

### Hyperdimensional computing (HDC)

In HDC, “hypervectors” (HVs), i.e., high-dimensional representations of data created from raw signals using an encoding procedure, constitute the basic building blocks of computational algorithms (Kanerva, 2009). These hypervectors are then combined and manipulated using specific mathematical operations (see below) to build transparent, symbolic computational models with the ability to preserve (memorize) the original information. Such memorization is enabled by a key property called “near-orthogonality". Consider two HVs ${\vec{H}}_{1},{\vec{H}}_{2}\in {\left\{-1,1\right\}}^{D}$ whose elements are independent and identically distributed (i.i.d.), each following the Rademacher distribution. If *D* is large enough (often *D* ~ 10^4^ in practice), these vectors become approximately orthogonal, as can be seen from their cosine similarity


$$\begin{array}{l} \delta \left(\vec{H_{1}},\vec{H_{2}}\right)=\frac{\vec{H_{1}}\cdot \vec{H_{2}}}{∥\vec{H_{1}}∥∥\vec{H_{2}}∥}≃0\;\text{if}\;D\gg 1 \end{array}$$

As such, (pseudo) random HVs with i.i.d. components are commonly used as essential ingredients in HDC encoding processes. Such HVs are then combined using established HDC operations to generate new HVs that have compositional characteristics and therefore allow computations to be performed in superposition, effectively encode spatial and temporal information, and respect intricate hierarchical relationships present in the data. The most commonly used HDC operations in the literature are as follows (Gayler, 1998; Zou et al., 2022; Kleyko et al., 2023):

**Binding (⊗):** Two HVs are bound together using component-wise multiplication of their elements. This operation is often used for creating association among HVs, is reversible (${\vec{H}}_{1}⊗\left({\vec{H}}_{1}⊗{\vec{H}}_{2}\right)={\vec{H}}_{2}$ and ${\vec{H}}_{2}⊗\left({\vec{H}}_{1}⊗{\vec{H}}_{2}\right)={\vec{H}}_{1}$), and the resulting HV can be shown to be nearly orthogonal to both operands ($\delta \left({\vec{H}}_{1}⊗{\vec{H}}_{2},{\vec{H}}_{1}\right)≃\delta \left({\vec{H}}_{1}⊗{\vec{H}}_{2},{\vec{H}}_{2}\right)≃0$).

**Bundling (+):** Two HVs are bundled together using component-wise addition of their elements. Unlike summation in small dimensions which results in an (irreversible) averaging, hyperdimensional bundling preserves the information of both operands. This can be seen from the fact that the bundled HV has non-negligible similarity with each of its operands ($\delta \left({\vec{H}}_{1}+{\vec{H}}_{2},{\vec{H}}_{1}\right)≃||{\vec{H}}_{1}|{|}^{2}\gg 0$ and $\delta \left({\vec{H}}_{1}+{\vec{H}}_{2},{\vec{H}}_{2}\right)≃||{\vec{H}}_{2}|{|}^{2}>>0$). Therefore, by performing a similarity check between a bundled HV and any query HV, one can determine whether the query has been one of the constituents of the bundled HV.

**Permutation (ρ):** Permutation is achieved by a circular shift of one HV's elements and is used to generate sequential order among HVs. We do not use permutation in the encoding of NeuroPixelHD.

### NeuroPixelHD encoding

**Receptive field encoding**. In this study, we adopted a novel approach to encode the identity of each neuron into one HV. Unlike using i.i.d. HVs for different neurons, this approach generates neuron HVs which are correlated with each other depending on the similarity between the receptive fields of their corresponding neurons. For this, we used the Gabor receptive field tuning experiments and computed the mean spike count of each neuron during the full 250ms presentation of each of the 81 Gabor locations, averaged over the 45 repetitions of each location.

This generates a (pre-encoding) 81-dimensional receptive field response vector ${\vec{F}}_{i}$ for each neuron *i*, which is then encoded into a *D*-dimensional HV via (Rahimi and Recht, 2007; Hernández-Cano et al., 2021).


(1) $$\begin{array}{ll} \text{Encoded receptive field response } & =\cos\left({\text{B}}^{T}{\vec{F}}_{i}+\vec{b}\right)⊗\sin\left({\text{B}}^{T}{\vec{F}}_{i}\right) \end{array}$$

where **B** is a 81-by-*D* random matrix with i.i.d. standard normal elements, $\vec{b}\in {\left[0,2\pi \right]}^{D}$ is a random vector with i.i.d. elements uniformly distributed over [0, 2π], and *D* = 10^4^ ≫ 81. This encoding is inspired by the Radial Basis Function (RBF) kernel trick and can account for nonlinear relationships among features during encoding.

**Brain area encoding**. In each of the subjects, spiking data from neurons in a subset of the following brain areas was available: VISp, VISam, VISal, VISrl, VISmma, VISpm, VISl, CA1, CA2, CA3, SUB, ProS, DG, TH, LP, LGv, LGd, PP, PIL, MGv, PO, Eth, POL, ZI, and APN. In principle, neurons in distinct areas can have very similar receptive field responses. Therefore, to further distinguish neurons from different areas, we define a unique, random and independent HV $\vec{R}\in {\left[0,1\right)}^{D}$ for each brain area. To simplify indexing notation, we use ${\vec{R}}_{i}$ to denote the area HV corresponding to each neuron *i*, thus ${\vec{R}}_{i}={\vec{R}}_{j}$ if neurons *i* and *j* belong to the same area. These area-specific HVs are then bound with encoded receptive field HVs, as described below (cf. Equation 2).

**Spiking activity encoding**. In each time bin, each neuron may have a zero or non-zero number of spikes. Both the occurrence and absence of spikes contains valuable information which need to be reflected in overall trial encoding. Motivated by our prior work (Zou et al., 2022), we define two *polarization* HVs ${\vec{H}}_{+}$ and ${\vec{H}}_{-}$, corresponding to the presence and lack of spikes, respectively. We generated ${\vec{H}}_{+}\in {\left\{\pm 1\right\}}^{D}$ with i.i.d. elements and let ${\vec{H}}_{-}=-{\vec{H}}_{+}$.

**Time encoding**. The duration of each trial, set at 250 milliseconds, is divided into *B* bins, *B* = 1, 2, 5, 10, 25, 50, 125, 250. For each time bin, *t* = 0, 1, …, *M* − 1, which *M* is the number of time hypervectors ($M=\frac{250}{B}$), a HV $\vec{T}\left(t\right)$ is constructed such that temporal correlation is maintained among ${\left\{T\left(t\right)\right\}}_{t=0}^{M}$.

This is achieved, independently for each trial, by generating random HVs $\vec{T}\left(0\right)$ and $\vec{T}\left(M-1\right)\in {\left\{0,1\right\}}^{D}$ for the initial and final time bins and linearly interpolating between them to generate time HVs for intermediate bins. Mathematically,


$$\begin{array}{l} \vec{T}\left(t\right)=\left(1-\frac{t}{M}\right)\vec{T}\left(0\right)+\frac{t}{M}\vec{T}\left(M-1\right) \end{array}$$

The resulting HVs retain temporal relationships depending on their temporal proximity.

**Trial encoding**. Finally, the HVs described earlier are combined through various levels of binding and bundling to generate a single HV encoding of each trial. This is done via


(2) $$\begin{array}{ll} {\vec{S}}_{i} & =\cos\left({\text{B}}^{T}{\vec{F}}_{i}+\vec{b}\right)⊗\sin\left({\text{B}}^{T}{\vec{F}}_{i}\right)⊗{\vec{R}}_{i} \end{array}$$

(3) $$\begin{array}{ll} {\vec{V}}^{k} & =\overset{M}{\underset{t=1}{\sum }}\left[\overset{N}{\underset{\begin{array}{c} i=1\\ n_{i}^{k}\left(t\right)\neq 0 \end{array}}{\sum }}n_{i}^{k}\left(t\right){\vec{S}}_{i}⊗{\vec{H}}_{+}+\overset{N}{\underset{\begin{array}{c} i=1\\ n_{i}^{k}\left(t\right)=0 \end{array}}{\sum }}{\vec{S}}_{i}⊗{\vec{H}}_{-}\right]⊗{\vec{T}}^{k}\left(t\right) \end{array}$$

The spatial HV ${\vec{S}}_{i}$ is the encoding (i.e., identity) of each neuron *i* and results from binding its encoded receptive field response in Equation (1) with its encoded area HV ${\vec{R}}_{i}$. These spatial HVs are then scaled and polarized appropriately, bundled over space, bound with corresponding time HVs, and then bundled over time to generate the final trial HV ${\vec{V}}^{k}$.

### NeuroPixelHD adaptive training

Following our earlier work (Zou et al., 2022), we employed an adaptive training approach that considers the *extent* to which each training data point is correctly or incorrectly classified in updating the class HVs. Consider a problem with *m* classes and *K*_train_ training samples (represented by encoded trial HVs) ${\left\{{\vec{V}}^{k}\right\}}_{k=1}^{K_{\text{train}}}$, where each training sample *k*∈*C*_*l*_ for some class *l* = 1, …, *m*. *C*_*l*_ denotes the set of all trial indices that belong to class *l*. The goal of the training is to generate one class HV ${\vec{C}}_{l}$ for each class *l* = 1, …, *m* such that each test sample ${\vec{V}}^{k}$ has the highest similarity with its own class HV, i.e.,


$$\begin{array}{l} k\in C_{\underset{1\leq l\leq m}{\mathrm{argmax}}\delta \left({\vec{V}}^{k},{\vec{C}}_{l}\right)}\;\text{for all test trials }k\text{.} \end{array}$$

All class HVs are initialized to zero at the beginning of training and gradually updated such that their similarity with training samples of their own class is increased and their similarity with training samples of other classes is decreased.

Consider first the case for the classification of natural scenes (*m* = 118). At initialization, all ${\vec{C}}_{l}$ are set to zero. Then, for each training sample ${\vec{V}}^{k}$, let ℓ denote its correct class (*k* ∈ *C*_ℓ_) and ℓ′ denote its predicted class ($\ell^{\prime }={\mathrm{argmax}}_{1\leq l\leq m}\delta \left({\vec{V}}^{k},{\vec{C}}_{l}\right)$). Further, define


$$\begin{array}{l} \delta_{\ell }=\delta \left({\vec{V}}^{k},{\vec{C}}_{\ell }\right),\;\delta_{\ell^{\prime }}=\delta \left({\vec{V}}^{k},{\vec{C}}_{\ell^{\prime }}\right). \end{array}$$

When the training sample is predicted correctly (ℓ′ = ℓ), the correct class HV ${\vec{C}}_{\ell }$ is updated in order to further increase its similarity with ${\vec{V}}^{k}$:


$$\begin{array}{l} {\vec{C}}_{\ell }={\vec{C}}_{\ell }+\eta \left(1-\delta_{\ell }\right){\vec{V}}^{k} \end{array}$$

The update is proportional to 1 − δ_ℓ_ so that ${\vec{C}}_{\ell }$ is modified less if its similarity with ${\vec{V}}^{k}$ is already high. If the training sample is predicted incorrectly (ℓ′ ≠ ℓ), the predicted class is also updated such that its similarity with ${\vec{V}}^{k}$ is decreased,


$$\begin{array}{ll} {\vec{C}}_{\ell } & ={\vec{C}}_{\ell }+\eta \left(\delta_{\ell^{\prime }}-\delta_{\ell }\right){\vec{V}}^{k}\\ {\vec{C}}_{\ell^{\prime }} & ={\vec{C}}_{\ell^{\prime }}-\eta \left(\delta_{\ell^{\prime }}-\delta_{\ell }\right){\vec{V}}^{k}. \end{array}$$

Similar to the previous case, the adaptive training considers the extent to which a training point is misclassified. In cases where the prediction is significantly off ($\delta_{\ell^{\prime }}\gg \delta_{\ell }$) the update equation substantially modifies ${\vec{C}}_{\ell^{\prime }}$, whereas for marginal mispredictions ($\delta_{\ell^{\prime }}≃\delta_{\ell }$), the update makes smaller adjustments. For both cases, we used η = 0.01 and performed the training for 3 epochs (rounds of presenting the training samples).

The above equations are slightly adjusted for the classification of the location of Gabor patches (*m* = 81) due to the presence of a natural notion of proximity between classes. In this case when the query data is predicted correctly (ℓ = ℓ′), we update not only the correct class HV ${\vec{C}}_{\ell }$ but also the HVs for the (up to 8) classes adjacent to it, i.e.,


$$\begin{array}{ll} {\vec{C}}_{\ell } & ={\vec{C}}_{\ell }+\eta_{center}\left(1-\delta_{\ell }\right){\vec{V}}^{k}\\ {\vec{C}}_{\nu } & ={\vec{C}}_{\nu }+\eta_{neighbor}\left(1-\delta_{\ell }\right){\vec{V}}^{k} \end{array}$$

for all classes (Gabor locations) ν adjacent to ℓ. Similarly, when each training sample is predicted incorrectly (ℓ′ ≠ ℓ), we let


$$\begin{array}{ll} {\vec{C}}_{\ell } & ={\vec{C}}_{\ell }+\eta_{center}\left(\delta_{\ell^{\prime }}-\delta_{\ell }\right){\vec{V}}^{k}\\ {\vec{C}}_{\nu } & ={\vec{C}}_{\nu }+\eta_{neighbor}\left(\delta_{\ell^{\prime }}-\delta_{\ell }\right){\vec{V}}^{k}\\ {\vec{C}}_{\ell^{\prime }} & ={\vec{C}}_{\ell^{\prime }}-\eta_{center}\left(\delta_{\ell^{\prime }}-\delta_{\ell }\right){\vec{V}}^{k}\\ {\vec{C}}_{\nu^{\prime }} & ={\vec{C}}_{\nu^{\prime }}-\eta_{neighbor}\left(\delta_{\ell^{\prime }}-\delta_{\ell }\right){\vec{V}}^{k} \end{array}$$

for all classes ν adjacent to ℓ and ν′ adjacent to ℓ′. We used η_*center*_ = 0.01 and η_*neighbor*_ = 0.001 and executed the algorithm for 2 epochs.

### Spatial averaging

In this study, we employed the HDC algorithm at various levels of spatiotemporal resolution. The spatial averaging involved five, progressively coarser levels: neuron level, population level, area level, region level, and whole brain.

At the neuron level (no spatial averaging), neurons were the basic spatial units, and we used the spike counts of all recorded neurons separately during the trial encoding process as summarized in Equations (2, 3).

At the population level, we clustered the neurons within each brain area into putatively excitatory (E) and inhibitory (I) populations. Among the various functional proxies suggested for E/I classification (Connors and Gutnick, 1990; Barthó et al., 2004; Becchetti et al., 2012), we used spike count Fano factor (variance-to-mean ratio), which was found by Becchetti et al. (2012) to most accurately distinguish the two populations in comparison with ground truth based on fluorescence imaging. For each neuron, its Fano factor was computed based on its number of spikes during a specific time bin across all Gabor positions and trials. This value is expected to be higher for inhibitory neurons than excitatory ones (Becchetti et al., 2012). Therefore, given the nominal 80-20 ratio of excitatory and inhibitory neurons (Beaulieu, 1993; Markram et al., 2004), we labeled the 20% of neurons in each area with highest Fano factor as putatively inhibitory and the rest as putatively excitatory (Supplementary Figure 1A). The spike counts of neurons within each E/I population were than summed and used instead of $n_{i}^{k}\left(t\right)$ in Equation (3), where *i* now refers to a population rather than a neuron. Accordingly, the spatial HVs ${\vec{S}}_{i}$ in Equation (2) were also replaced by population HVs computed via binding a randomly generated E/I HV (same across all regions) with the corresponding area HV ${\vec{R}}_{i}$. Considering the potential for the above clustering based on Fano factor to produce varied clusters depending on the chosen time resolution, particularly for neurons that exhibit intermediate traits, we compared the classification accuracy of population-level classifiers that used different bin sizes in the computation of Fano factors, and selected the Fano factor bin size that achieved the highest classification accuracy for each mouse. The resulting optimal population-level model was then compared against other spatial resolutions at the finest (1ms) temporal resolution.

At the area level, the spike counts of all neurons within each area were summed and used instead of $n_{i}^{k}\left(t\right)$ in Equation (3). Spatial HVs ${\vec{S}}_{i}$ in Equation (3) were accordingly replaced by ${\vec{R}}_{i}$. Similarly, at the region level, the spike counts of all neurons within each region were summed and used instead of $n_{i}^{k}\left(t\right)$ in Equation (3). Table 1 shows the assignment (clustering) of brain areas to regions. For each region, one random and independent spatial HV was generated and used as ${\vec{S}}_{i}$ in Equation (3). Finally, at the whole-brain level, the spike counts of all recorded neurons for each mouse were combined, simplifying Equations (2, 3) to ${\vec{V}}^{k}=\overset{M}{\underset{t=1}{\sum }}n^{k}\left(t\right){\vec{T}}^{k}\left(t\right)$.

### Temporal averaging

To assess the optimal temporal resolution for visual decoding, we binned raw spike counts into bin sizes of 1, 2, 5, 10, 25, 50, 125, 250 ms, effectively averaging spike counts at the finest scale (1ms) over larger bins. The resulting binned spike counts are provided to the NeuroPixelHD encoder, i.e., $s_{i}^{k}\left(t\right)$ in Equation (3).

### Data augmentation

The amount of neural data available to train the NeuroPixelHD classifier is relatively small compared to contemporary machine learning experiments, even though it consists of one of the largest MUA datasets available to date. In particular, the number of trials in which the exact same stimulus is shown to the mice (45 for Gabor patches and 50 for natural images) allows for no more than 1–2 dozen test samples per class, which often results in low statistical power when comparing among different spatiotemporal resolutions. This is often treated with data augmentation, for which various techniques have been proposed (Antoniou et al., 2017; Shorten and Khoshgoftaar, 2019; Bayer et al., 2022). In this work we used a novel form of data augmentation for comparisons between different spatial and temporal scales which exploits the specific dynamical structure of our data. Let the three-dimensional array **N**_*N*×*T*×*K*_ contain all the binned spike counts of *N* neurons over *T* time bins and *K* trials of the same class (same image). Then, we randomly shuffle the trial indices, uniformly for all neurons and independently for all times. In other words, we generate *T* independent sets of random indices $\left(j_{1}^{t},\ldots ,j_{K}^{t}\right),t=1,\ldots ,T$ each of which is a permutation of (1, …, *K*), and generate a permuted array $\hat{\text{N}}$ where


$$\begin{array}{l} \hat{\text{N}}\left(:,t,k\right)=\text{N}\left(:,t,j_{k}^{t}\right),\;\text{for all }t,k. \end{array}$$

This process is repeated four times for each class, separately among training and test samples, resulting in a 5-fold increase in the total number of training and test samples.

### Measures of classification accuracy

For classifiers trained on images of natural scenes (118 images, each serving as one classification category), we measured their accuracy using cross-validated F1 score,


$$\begin{array}{l} F_{1}=2\frac{\text{precision}\cdot \text{recall}}{\text{precision}+\text{recall}} \end{array}$$

where $\text{precision}=\frac{\text{true positive}}{\text{true positive}+\text{false positive}}$ and $\text{recall}=\frac{\text{true positive}}{\text{true positive}+\text{false negative}}$. This was computed for each class using sklearn.metrics.precision_recall_fscore_support in python. The F1 score ranges from 0 to 1, with higher values indicating better classification and $F_{1}=\frac{1}{118}=0.008$ representing chance level.

For classifiers trained on the location of Gabor patches, we incorporated the geometric nature of the task and instead used the distribution of Euclidean distances between the true location and the prediction location of each Gabor patch,


$$\begin{array}{l} \text{Euclidean error}=\sqrt{{\left(x_{\text{true}}-x_{\text{pred}}\right)}^{2}+{\left(y_{\text{true}}-y_{\text{pred}}\right)}^{2}},\\ \left(x_{\text{true}},y_{\text{true}}\right),\left(x_{\text{pred}},y_{\text{pred}}\right)\in {\left\{0,1,\ldots ,8\right\}}^{2}. \end{array}$$

This metric distinguishes between slight misclassifications, where the predicted location is close to the true one, and large misclassifications where the predicted location is many cells away from the true one. Given that the patches were presented at either cell of a 9x9 grid, each Euclidean distance can range from 0 to $8\sqrt{2}$, with a chance level of approximately 4.7.

### Alternative classifiers

Alternative machine learning classifiers were implemented using the scikit-learn package version 1.3.0 in python with the following parameters. Random forest: 10 estimators; k-nearest-neighbor: 18 neighbors; artificial neural network: multi-layer perceptron with one hidden layer, 10 hidden units, and ReLU activation. We also experimented with support vector machine (SVM) classification due to its conceptual similarity with HDC but had to remove it from comparisons due to its infeasibly high run times. For all algorithms, each training/test sample consisted of binned spike counts from all neurons throughout one trial at some specific spatial and temporal resolution. In other words, for each trial, a dataframe was created in which rows represent spatially averaged units of neuronal response (including individual neurons if the algorithm is applied at the neuron-level spatial resolution), columns represent time bins, and values represent binned spike counts at the corresponding spatiotemporal resolution. This dataframe was then flattened (vectorized) and used as input feature for all algorithms.

### Estimating the central frequency of neural oscillations

In order to estimate the central oscillatory frequency of each brain region illustrated in Figure 3B, we computed each region's average firing rate via averaging the 1ms-binned spike counts of all neurons within that region. The resulting time series was not broken or averaged across trials but was rather estimated as one contiguous stream throughout all Gabor classification trials. For each region, power spectral density was then computed using the Welch's method and passed through the FOOOF toolbox (Donoghue et al., 2020) to estimate the central frequency of the slowest neural oscillation. We manually inspected FOOOF estimates and adjusted its hyper-parameters to ensure accuracy of its findings. We discarded oscillations below 3Hz due to lack of sufficient frequency resolution to determine whether an oscillation actually existed in this range. For regions that showed multiple oscillations above 3Hz, we only selected the slowest one. In the vast majority of cases, these lied in the theta range. In very few cases, no theta oscillations were detectable, in which case we recorded no oscillation for that region (instead of, e.g., recording a gamma-band oscillation which would have caused inconsistency with other regions).

### Statistical testing

All statistical testing was performed using the non-parametric Wilcoxon signed rank test. The only exception is the testing of the significance of the Pearson correlation coefficient between optimal temporal resolution and oscillatory central frequency in Figure 3B. For the latter, we used non-parametric randomization testing with 10^4^ random permutations.

### Computing

All the computations reported in this study were performed on a Lenovo P620 workstation with AMD 3970X 32-Core processor, Nvidia GeForce RTX 2080 GPU, and 512GB of RAM.

## Data availability statement

The original contributions presented in the study are included in the article/Supplementary material, further inquiries can be directed to the corresponding author.

## Ethics statement

The animal study was approved by the Allen College Institutional Review Board (ACIRB). The study was conducted in accordance with the local legislation and institutional requirements.

## Author contributions

TS: Conceptualization, Data curation, Investigation, Methodology, Software, Writing – original draft, Formal analysis. ZZ: Writing – original draft, Methodology. MI: Methodology, Supervision, Writing – review & editing, Conceptualization, Project administration. EN: Conceptualization, Formal analysis, Supervision, Validation, Writing – original draft, Data curation, Funding acquisition, Investigation, Methodology, Project administration, Resources.
